# Supplementary material for: WordCommentsAnalyzer: A windows software tool for qualitative research
Source: F1000Res. 2018 Sep 4;7:536. Originally published 2018 May 3. [Version 2] doi: 10.12688/f1000research.14819.2 (PMC6137411; doi:10.12688/f1000research.14819.2)
Supplement: Supplementary file 3 [file f1000research-7-17614-s0002.tgz › b2a79496-4ada-47c4-9682-a99ce867836d.zip!/Eight Iranian Health Journals/Archives of Rehabilitation_3.docx]

© Archives of Rehabilitation (RJ). University of Social Welfare and Rehabilitation Sciences, Koodakyar Alley, Daneshjoo Blv., Evin, Tehran, Iran. This work is licensed under [CC BY-NC 4.0](https://creativecommons.org/licenses/by-nc/4.0/).

Volume & Issue: دوره 18 - شماره 3

Title: عوامل مؤثر بر پذیرش افراد دارای ناتوانی در جامعه (مطالعه موردی: شهرکرد)

http://rehabilitationj.uswr.ac.ir/article-1-1872-fa.html

هدف افراد دارای ناتوانی باید از نظر جسمانی و روانی حمایت شوند و تحقیقات بیشتری برای حل مشکلات آن‌ها انجام شود. پژوهش حاضر به بررسی عوامل مؤثر بر پذیرش این افراد در جامعه می‌پردازد. روش بررسی روش پژوهش در این مقاله پیمایشی است و جامعه آماری شامل 4هزار و 58 نفر افراد دارای ناتوانی تحت پوشش سازمان بهزستی شهرکرد ( 2هزار و 474 نفر دارای ناتوانی جسمی‌حرکتی، 673 نفر دارای ناتوانی بینایی، 911 نفر دارای ناتوانی شنوایی) می‌شود که با روش کوکران تعداد نمونه آماری برابر با 352 نفر است. بر اساس روش نمونه‌گیری سهمیه‌ای 58 نفر نابینا،80 نفر ناشنوا و 214 نفر افراد دارای ناتوانی جسمی‌حرکتیبودند که به صورت تصادفی انتخاب شدند. ابزار جمع‌آوری اطلاعات پرسش‌نامه محقق‌ساخته بود. برای اعتبار و روایی پرسش‌نامه از روش اعتبار صوری و آلفای کرونباخ استفاده شد. استاد راهنما، مشاوران و استادان مجرب پرسش‌نامه را تأیید کردند و با استفاده از روش کرونباخ مقدار آلفا برای متغیر پذیرش افراد دارای ناتوانی 82/0، سرمایه اجتماعی 86/0، حمایت خانواده 94/0، نحوه گذراندن اوقات فراغت 82/0، ارائه خدمات توان‌بخشی 93/0، امکانات رفت‌وآمد 85/0، برنامه‌های رسانه‌های جمعی 79/0، ویژگی‌های شخصیتی 92/0 و آموزش و کارآفرینی 87/0 است . یافته‌ها نتایج آزمون ضریب همبستگی پیرسون نشان داد بین متغیرهای سرمایه اجتماعی، امکانات رفت‌وآمد، نحوه گذراندن اوقات فراغت، ارائه خدمات توان‌بخشی، آموزش وکارآفرینی، برنامه‌های رسانه‌های جمعی، ویژگی‌های شخصیتی، حمایت خانواده با پذیرش افراد دارای ناتوانی همبستگی معنا‌داری وجود دارد (001/0(P<. بین متغیرهای نوع (108/0P=)، سابقه (531/0P=) و علت ناتوانی (427/0P=) با پذیرش افراد دارای ناتوانی رابطه معناداری وجود ندارد. بر اساس نتایج آزمون‌های تحلیل واریانس و آزمون تی بر حسب مشخصات جمعیت‌شناختی افراد دارای ناتوانی نشان داده شد که بین جنسیت (071/0P=) و سن (498/0P=) و پذیرش افراد دارای ناتوانی در جامعه تفاوت معناداری وجود ندارد و بین تحصیلات، تحصیلات پدر و شغل افراد دارای ناتوانی و پذیرش آن‌ها در جامعه تفاوت معناداری وجود دارد (001/0P<). افراد دارای تحصیلات بالاتر میانگین پذیرش بیشتری در جامعه دارند و همچنین میانگین پذیرش گروه‌های شغلی پزشک و پرستار، کارمندان فنی و بازاری‌ها در جامعه بالاتر است. همچنین بین وضعیت تأهل (032/0P=) و تحصیلات همسر (046/0P=) و پذیرش افراد دارای ناتوانی نیز تفاوت معناداری وجود دارد. مهم‌ترین متغیرهای مؤثر در پذیرش افراد دارای ناتوانی سرمایه اجتماعی (240/0B=)، خانواده (198/0B=)، نوع ناتوانی (164/0B=)، برنامه‌های رسانه‌های جمعی (163/0B=)، امکانات رفت‌وآمد (159/0B=) و کارآفرینی و آموزش (129/0B=) است.  نتیجه‌گیری پذیرش افراد دارای ناتوانی برای حضور در جامعه و روابط اجتماعی لازم است. با ایجاد امکانات و شرایط مناسب نظیر امکانات آموزشی، رفت‌وآمد، اوقات فراغت و مانند این‌ها و حمایت بیشتر از این افراد فرصت حضور آن‌ها در جامعه بیشتر می‌شود و زندگی آنان بهبود می‌یابد.

Title: اثر فعالیت شناختی بر تعادل راه‌رفتن در افراد مبتلا به بی‌ثباتی عملکردی مچ پا

http://rehabilitationj.uswr.ac.ir/article-1-1956-fa.html

هدف گروهی از افراد مبتلا به بی‌ثباتی مزمن مچ پا، با وجود اینکه علائمی از سستی لیگامان‌های خارجی مفصل را نشان نمی‌دهند، از کاهش عملکرد و خالی‌شدن مفصل مچ پا به‌ویژه حین انجام فعالیت‌های دینامیک رنج می‌برند که از آن با عنوان بی‌ثباتی عملکردی مچ پا یاد می‌شود. سازماندهی اطلاعات دریافتی از سیستم‌های حسی و به دنبال آن تولید پاسخ‌های حرکتی مناسب و نیز تعدیل استراتژی‌های مورد نیاز برای تطبیق موقعیت بدن نسبت به شرایط محیطی، نیازمند فعالیت سیستم‌های شناختی و توجه است. مطالعات اندکی به بررسی تأثیر فعالیت شناختی بر کنترل پوسچرال افراد مبتلا به بی‌ثباتی عملکردی مچ پا پرداخته‌اند. این مطالعات قابل تعمیم به شرایط دینامیک راه‌رفتن به عنوان یکی از مهم‌ترین و پراستفاده‌ترین فعالیت‌های روزانه نیست. اعمال تکلیف شناختی می‌تواند باعث ایجاد تغییراتی در روند راه‌رفتن و تعادل شود که ممکن است احتمال خالی‌شدن مفصل مچ پا را افزایش دهد. این مطالعه با هدف بررسی تأثیر فعالیت شناختی بر تعادل حین راه‌رفتن در افراد مبتلا به بی‌ثباتی عملکردی مچ پا انجام شده است. روش بررسی به منظور انجام این پژوهش 12 فرد آماده از نظر جسمانی که به بی‌ثباتی عملکردی مچ پا مبتلا بودند و 12 فرد سالم انتخاب شدند. برای هر فرد سه مرحله ارزیابی انجام شد که عبارت بود از: راه‌رفتن با سرعت عادی و راه‌رفتن همراه با اعمال تکلیف شناختی و اعمال تکلیف شناختی در وضعیت نشسته. برای انجام تکلیف شناختی از افراد خواسته شد هم‌زمان با راه‌رفتن از رقمی که به صورت تصادفی اعلام می‌شود (اعداد بین 200 تا 250؛ ارقامی که به 0 یا 7 ختم می‌شوند حذف شدند)، 7 تا 7 تا به عقب برگردند. داده‌ها با استفاده از هفت دوربین مادون قرمز ثبت و با نرم‌افزار Visual-3D محاسبه شد. متغیرهای سرعت راه‌رفتن، زمان و طول قدم، زمان فاز استانس و سوئینگ محاسبه شد. برای مقایسه متغیرها قبل و بعد از تحمیل تکلیف شناختی از آزمون پارامتریک تی زوج و برای مقایسه نتایج بین گروه مبتلا و کنترل از آزمون تی مستقل استفاده شد. تغییرپذیری زمان قدم با استفاده از ضریب همبستگی درون‌گروهی محاسبه شد.  یافته‌ها افراد مبتلا به بی‌ثباتی عملکردی مچ پا در سرعت قدم کاهش معنادار و در زمان فاز استانس و سوئینگ و زمان قدم طی اعمال تکلیف شناختی نسبت به شرایط بدون اعمال تکلیف شناختی افزایش معناداری نشان دادند (05/0P<). افراد مبتلا، تغییرپذیری بیشتری را در هر دو حالت راه‌رفتن و در متغیر زمان قدم نسبت به گروه سالم نشان دادند.  نتیجه‌گیری با توجه به نتایج به‌دست‌آمده مشخص شد افراد مبتلا به بی‌ثباتی عملکردی مچ پا در وضعیت تکلیف دوگانه زمان بیشتری را در فاز سوئینگ سپری می‌کنند و در هر دو حالت تکلیف یگانه و تکلیف دوگانه نسبت به افراد سالم ثبات دینامیک کمتری دارند. استراتژی حرکتی متفاوت افراد مبتلا به بی‌ثباتی عملکردی مچ پا در شرایط تکلیف دوگانه نسبت به افراد سالم می‌تواند خطر پیچ‌خوردگی مجدد را افزایش دهد.
